# Impact of circuit training structures on the acute response in physiological and mechanical performance: a cross-sectional study

**DOI:** 10.1038/s41598-025-08432-1

**Published:** 2025-07-03

**Authors:** Francisco Hermosilla-Perona, Adrián Martín-Castellanos, Marcos Raphael Pereira-Monteiro, Javier Iglesias García, Manuel Barba-Ruíz, Juan Ramón Heredia-Elvar

**Affiliations:** 1 AExPH. Facultad de Ciencias Biomédicas y de la Salud., Universidad Alfonso X El Sabio, 28691 Madrid, Spain; 2 Facultad de Ciencias de la Vida y la Naturaleza, Universidad Nebrija, 28015 Madrid, Spain; 3 Facultad de Ciencias de la Actividad Física y del Deporte (INEF—Sports Department), Universidad Politécnica de Madrid, 28040 Madrid, Spain; 4 Graduate Program in Physiological Sciences, Federal University of Sergipe, São Cristóvão, Sergipe 49100-000 Brazil

**Keywords:** Lactate, Circuit training, Heart rate, Countermovement jump, Performance, Training configurations, Physiology, Public health

## Abstract

This study analyzed physiological and mechanical responses to different circuit resistance training structures in young adults. A descriptive cross-sectional study was conducted to evaluate the acute effects of four distinct circuit resistance training protocols on blood lactate, heart rate, and countermovement jump (CMJ) mechanical variables in 30 experienced young adults. The training sessions differed in both exercise order (alternated vs. grouped) and training volume (maximal vs. submaximal repetitions): A1 (alternated, maximal), G1 (grouped, maximal), A2 (alternated, submaximal), and G2 (grouped, submaximal). Each protocol included upper and lower limb exercises performed on guided machines with standardized rest intervals. Mechanical properties of the CMJ were assessed before and after each session, lactate concentrations were measured pre-, mid-, and post-exercise, and heart rate was continuously monitored throughout all sessions. Results showed no significant differences in CMJ height between groups; however, power-related variables experienced greater declines in the high-volume protocols (A1 and G1). The rate of force development was also more negatively affected in these higher-volume conditions. Mid-session blood lactate levels differed significantly across groups, particularly between high- and low-volume protocols, although no differences were observed at the end of the sessions. Heart rate varied significantly between the 4th and 12th minute of exercise, reflecting the influence of volume rather than exercise order. In conclusion, training volume appears to be the primary factor influencing both physiological and mechanical responses during circuit resistance training, rather than the specific configuration of the exercises.

## Introduction

Resistance training is widely recognized for its positive effects on the health of adults^1^, older adults^2^ and children^3^ and it is also considered essential in the sports context^4^. It is common for classic training approaches to perform several sets of the same exercise consecutively, popularly known as horizontal progressions. On the other hand, in vertical progressions different exercises were performed to complete each set, for instance, circuit structures stand out with this premise^5^. Among the session configurations widely used to apply resistance training, circuit structures stand out with effects on strength and power in adults^6^ as well as being seen as a practical option in a society with limited time for exercise^7^.

The acute effects generated by the circuit format provide adaptations in cardiovascular fitness^8,9^, causing better tolerance to greater cardiovascular demands in the long term. Regarding this, blood lactate concentration is an important physiological marker, which has higher concentrations in situations of greater anaerobic metabolism stress, caused by higher intensities^10^. Lactate is associated with other commonly used marker of intensity, the heart rate^11^, being both characterized as classical internals load variables used to monitor exercise^12^. In this sense, exercise protocols with greater intensity tend to increase plasma lactate concentrations and reduce neuromuscular responsiveness as the lower limb muscle power, measured by the countermovement jump (CMJ) height and its mechanical properties^13^.

Despite the advantages, controlling variables related to training volume in terms of number of repetitions and sets, and intensity referring to absolute load (e.g., kilograms or Newtons) and relative load (number of repetitions performed in each One Maximal Repetition percentage) in a circuit-based model remains challenging. Nuñez et al.^14^ explored the effects of different session configurations on blood lactate and heart rate. However, their study used endurance training circuit configurations in addition to resistance training, exploring the interference effect, but not providing information about different resistance training models^14^. Specifically about resistance training models, the studies in circuit format commonly establish the order of their exercises by grouping them according to some initial premise, such as motor pattern^15,16^ or target muscle^17^. In this scenario, Corrêa-Neto et al.^18^ stands out for verifying the acute impact of varying the order of exercises in circuit models of resistance training on fatigue-related aspects, presenting responses in blood pressure, perceived exertion, and volume measurements. Nevertheless, the article does not explore directly measurable mechanical variables and classic physiological markers such as blood lactate concentration and heart rate. Moreover, it is worth pointing out that the protocol did not present a proposal for full-body training, not incorporating exercises into a complete upper limb routine^18^.

Although the literature points to interesting responses to training based on the order of exercise, the acute effects of order based on upper and lower limb divisions, which are commonly used in professional practice, are unknown. As such, it is not clear how the internal load variables behave in these different session configurations. In this sense, this study aimed to analyse the physiological and mechanical responses to different structures of circuit resistance training in young adults. Our initial hypothesis was that circuit resistance training structures with maximal volume would lead to a greater impact in physiological and mechanical variables related to performance compared to structures with submaximal volume. The same rationale was applied to the comparison between grouped and alternated structures.

## Methodology

### Experimental design

A descriptive cross-sectional study was conducted. A total of four experimental sessions were planned for data collection and one session for familiarization, in order to analyze the acute impact of different training circuits on lactate, heart rate, jump height and mechanical variables of the CMJ.

### Participants

The study was conducted with a sample of 30 young adults (21 females, 9 males; 67.93 ± 14.14 kg; 1.60 ± 0.32 m; 22.48 ± 4.58 years), all of whom had at least 1 year of experience in resistance training. Participants were recruited through a public announcement posted in university sports science departments and affiliated fitness centers, inviting voluntary participation in the study. Inclusion criteria required participants to be between 18 and 30 years old, to have prior experience in resistance training, and to be free from musculoskeletal injuries during the 6 months preceding the study. Individuals were excluded if they reported any cardiovascular, neurological, or metabolic conditions that could interfere with exercise performance or pose a health risk. None of the participants reported any injuries during the study period or in the 6 months preceding the research.

The sample size was calculated using G*Power software (version 3.1.9.2, Kiel, Germany). In the preliminary analysis, it was determined that with an alpha of 0.05 and a power of 80%, 27 participants were required. Subsequently, based on the estimated effect size, (ƒ = 0.83; ANOVA with repeated measures, within-between interaction) with an alpha of 0.05 and a power of 95%, the statistical power of the analysis was calculated at 0.997. All participants completed the study without dropouts. Before participating, the participants were fully briefed on the experimental procedures and informed about the potential risks. Written informed consent was obtained from all participants before their involvement in the study. Data collection was carried out in accordance with the Declaration of Helsinki, in order to preserve human rights and to protect the privacy of the participants^19^. The study protocol was approved by the Ethics Committee of the Universidad Alfonso X el Sabio (2024_12/312).

### Procedure

In the study design, four measurements were conducted, each separated by 48–72 h. The measurements were organized into alternating and grouped exercise arrangements, considering the order of the exercises^17^. The alternating circuit (A1) involved a mixed order of six upper and lower limb exercises, while the grouped circuit (G1) concentrated on performing the three upper limb exercises first, followed by the three lower limb exercises. In all measurements, the exercises performed were consistent: three upper limb exercises (bench press, military press, and bilateral rowing) and three lower limb exercises (leg press, leg curl, and knee extension). All exercises were performed using guided machines to facilitate execution and load adaptation. During each measurement, participants completed two laps of the circuit involving these six exercises. The external load used for each exercise was established during the familiarization session based on the participant’s capacity to perform 10–12 repetitions with proper technique. This same load was maintained across all four training sessions (A1, G1, A2, G2) to ensure consistency in the intensity performed. The only variation between protocols was the number of repetitions performed: in A1 and G1, participants completed maximal repetitions to failure; in A2 and G2, they performed approximately 50% of their maximum repetition capacity during the first lap.

**Fig. 1:**
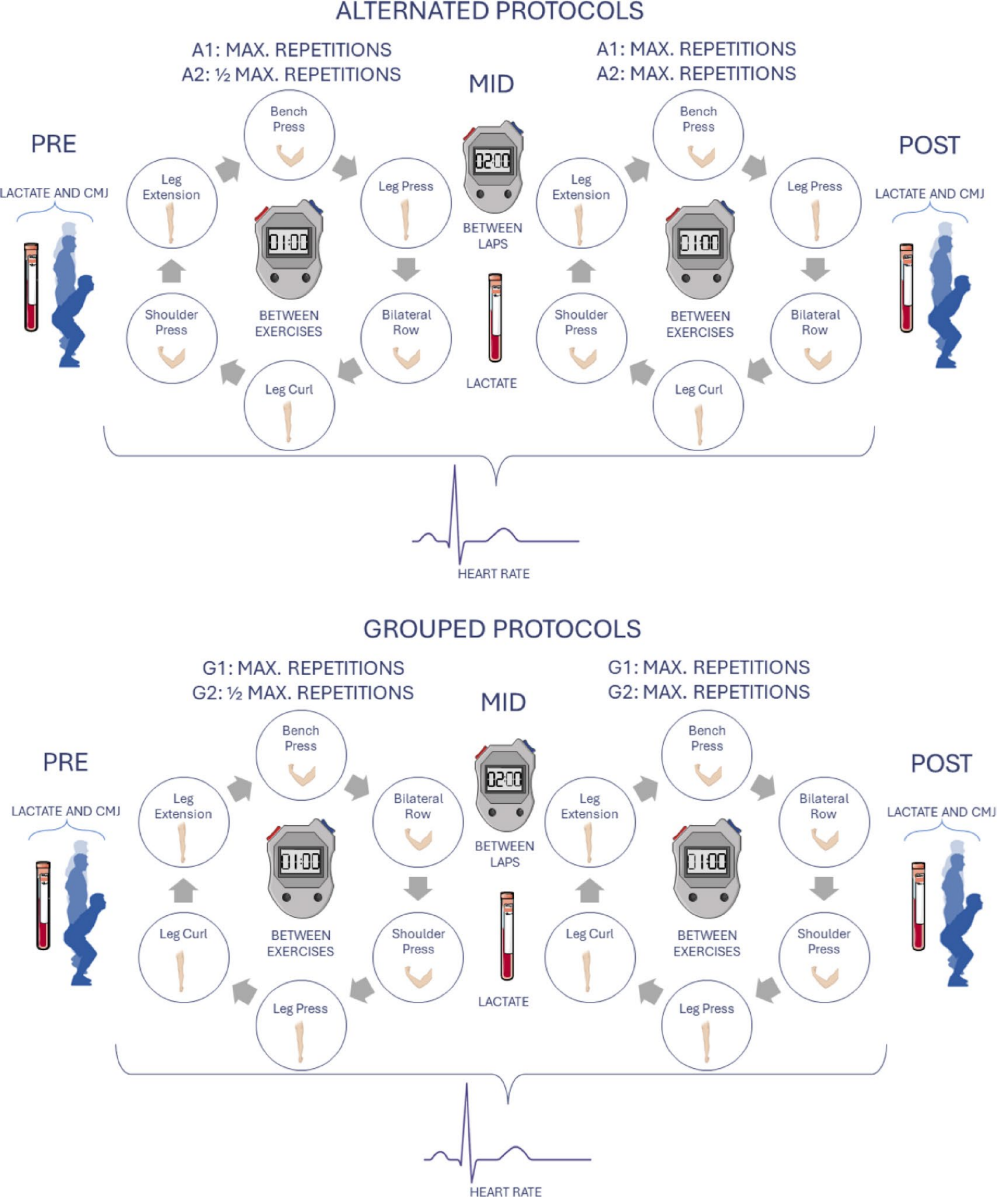
Protocol of exercises performed and measurements carried out.

In the first and second sessions, was performed alternating (A1) and grouped (G1) circuits, respectively. In A1 and G1 participants were required to perform the maximum number of repetitions in both laps for all exercises. However, in the third and fourth sessions (A2 and G2, respectively), participants performed half the number of repetitions in the first lap as they did in first lap of A1 and G1, but in the second lap, they were required to perform as many repetitions as possible. The rest periods for each exercise and between laps were standardized: participants rested for 1 min between exercises and 2 min between laps. The protocol of procedures is explained in Fig. 1.

The mechanical variables for this study were collected Pre and Post intervention. Specifically, were measured the height of CMJ, and maximal force (N/Bw), absolute maximal force (N), mean force (N), push impulse (N*s), positive push impulse (N*s), relative maximal power (W/Bw), absolute maximal power (W), mean power (W) and the maximum value for rate of force development, during the CMJ. For graphic aims, normalised time was computed between 0% at the start of the test and 100% at the take-off of the participants, data were normalized using linear interpolation. Jump measurements were conducted using the Quattro Jump 9290BA force plate (Kistler, Winterthur, Switzerland). Data was recorded at a sampling frequency of 500 Hz.

The blood lactate concentration was taken in three different moments: Pre, Mid and Post-intervention. After cleaning the skin, a blood capillary sample was collected using fingertip punctures on the middle finger. Lactate was measured using a portable lactate analyser (Lactate Pro 2, Arkray, Kyoto, Japan). The heart rate was recorded using a Polar heart rate meter (Verity, POLAR, Kempele, Finland) at every 30 s during all the exercise sessions.

### Statistical analysis

To verify the effects of structures of the circuit (A1, G1, A2, G2) and time (Pre and Post) on the CMJ variables (outcomes) we performed mixed models with subsequent Bonferroni post hoc analysis. Specifically, regarding the distribution, we used the Gamma distribution, because this model fitted better than the Gaussian distribution based on the Akaike Information Criterion. When the Gamma distribution was not applicable (when data contained neutral and/or negative values), we utilized a Gaussian distribution. We used the groups and time as factors to analyze the biomechanical variables as dependent variables. We include the participants as a cluster variable in the model as a random effect on intercept to contemplate the variability of any individual. Regarding Lactate, the normality of the data was verified using the Shapiro–Wilk test Once the data was considered normal, we conducted a repeated measures analysis of variance (ANOVA 4 × 3) to compare the dependent variables in different groups (A1, A2, G1, G2) over time (Pre, Mid and Post), with a subsequent Bonferroni post hoc analysis. Additionally, a One-Factor ANOVA was carried out to compare the percentual loss of jump height between the groups. The effect sizes (ES) were determined using partial eta squared (η_p_^2^) and their interpretation was based on the following criteria^20^: 0.01 ≤ ES < 0.06 indicates small effects, 0.06 ≤ ES < 0.14 indicates moderate effects, and ES ≥ 0.14 indicates large effects.

In turn, considering that we measured heart rate every 30 s, averages were calculated between time intervals (every four measurements) to compare the models and also repeated measures analysis of variance with Bonferroni post hoc. In addition, we calculated the Cohen’s d ES with a proposed correction by Rhea, thus establishing values for recreationally trained people, with values < 0.35 considered trivial, between 0.35 and 0.80 considered small; between 0.80 and 1.50 considered moderate; and above 1.5 considered large^21^. We consider *p* values below 0.05 to be statistically significant. We conducted all analyses using GraphPad Prism (version 10.0.0 for Windows, GraphPad Software, Boston, Massachusetts USA, www.graphpad.com) and Jamovi (The Jamovi Project, Jamovi, Version 2.4.11)^22,23^.

## Results

**Fig. 2:**
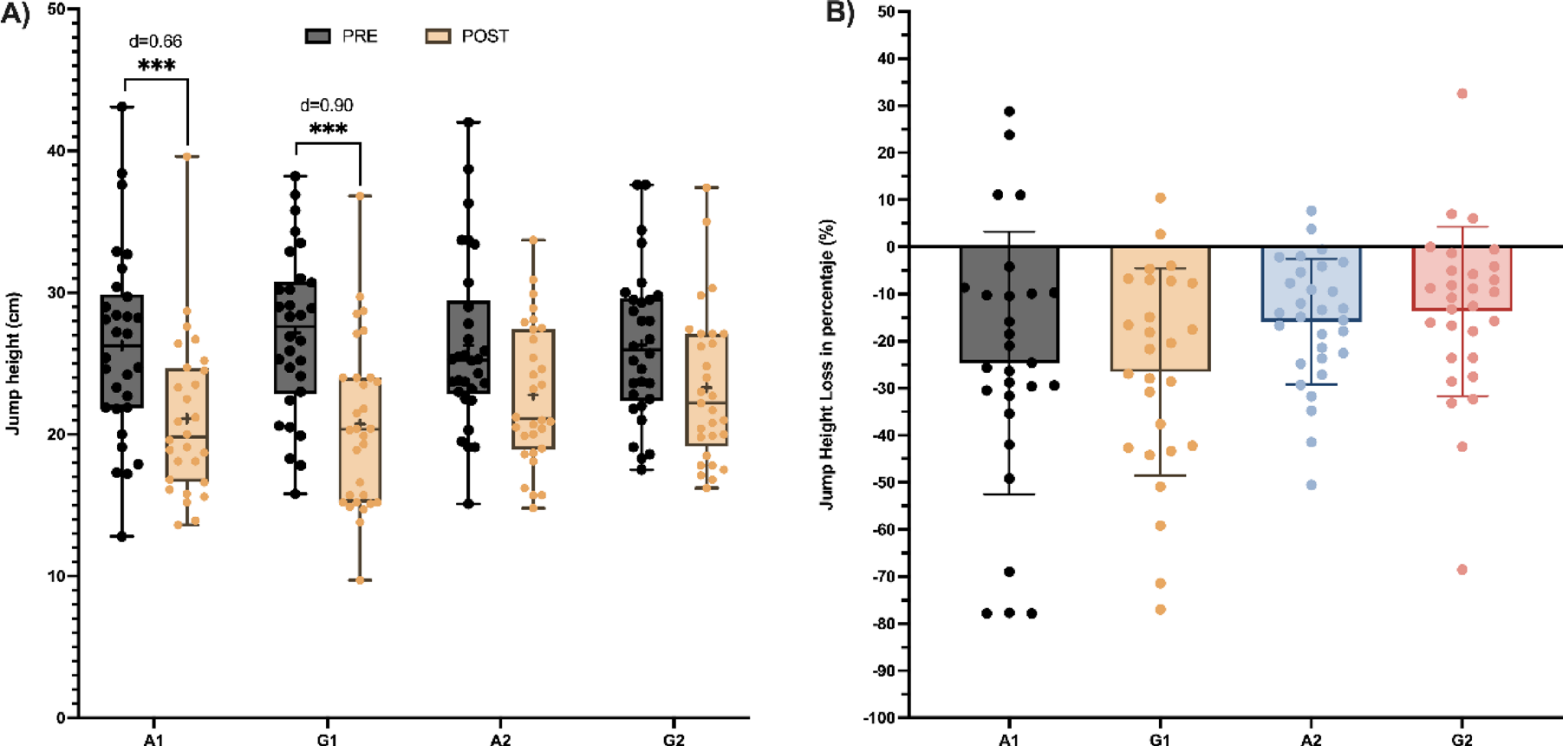
Changes in CMJ Height A: Comparisons Pre-post. B: Comparisons of percentual change. Note: A1: Alternated 1; A2: Alternated 2; G1: Grouped 1; G2: Grouped 2; Sig Codes: ***p < .001.

A mixed model analysis identified an interaction effect between group and time for the CMJ height (*X*^2^ = 11.96; *p* = .007). A significant reduction in post-intervention for the groups with maximal repetitions (A1 and G1) was reported, However, no statistical differences were found between pre and post-measurements in the groups A2 and G2 (Fig. 2A). Additionally, we identified a time effect (*X*^2^ = 60.72; *p* ≤ .001), but not a group effect (*X*^2^ = 3.42; *p* = .331)..

The magnitude of the decrease between both conditions was similar (Fig. 2B). In conditions with similar volume during the first round, a similar pattern was observed in the percentage decrease in jump height. Descriptive results indicated that conditions with higher volume (A1 and G1) exhibited higher percentages of decrease compared to A2 and G2, though no statistical differences were observed (*F*_(3, 110)_ = 2.63;* p* = .055).

Regarding CMJ mechanical variables, a mixed model analysis found an interaction effect between group and measurement time was found for push impulse (PI), relative maximal power (RMP), absolute maximal power (AMP), mean power (MP), and maximum rate of force development (RFDmax). Table 1 illustrates that groups A1 and G1 obtained lower Post values for PI and RMP with higher values than A2 and G2 (*p* < .05). AMP present significant differences between pre–post in all interventions (*p* < .05), except for A2. Also, post-values were significant lower in A1 and G2 than A2 and G2 respectively (*p* < .05). Finally, MP and RFDmax decline after the intervention in all groups, however A2 and G2 present higher post-values than A1 and G1 (*p* < .05). No interaction effect was found for relative maximal force (RMF), absolute maximal force (AMF), mean force (MF), and positive push impulse (PPI), though pre-values.

**Table 1:** Mechanical variables related to CMJ considering time and group.

	A1		G1		A2		G2		X^2^ (*P* inter)	X^2^ (*P* time)	X^2^ (*P* group)
	Pre	Post	Pre	Post	Pre	Post	Pre	Post			
RMF (N/Bw)	215 ± 25.5	205 ± 21.3*	215 ± 19.7	209 ± 22	225 ± 25.3	213 ± 20.5	225 ± 28.3	214 ± 25.5	1.09 (.779)	29.80 (< .001)	12.18 (.007)
AMF (N)	1386 ± 289	1329 ± 304*	1426 ± 370^§^	1385 ± 359	1489 ± 359	1403 ± 350*	1492 ± 333^Ψ^	1429 ± 337*	2.62 (.454)	36.23 (< .001)	20.45 (< .001)
MF (N)	1110 ± 262	1036 ± 233*^†^	1154 ± 303	1100 ± 283*	1179 ± 300	1131 ± 285^#^	1185 ± 299	1132 ± 288*	5.42 (.144)	49.00 (< .001)	29.79 (< .001)
PPI (N*s)	200 ± 45.8	171 ± 40.7*^†^	205 ± 51.4	184 ± 51.6*^§^	212 ± 50.8	197 ± 51.7*	216 ± 50.7	199 ± 51.7*	4.72 (.193)	78.98 (< .001)	29.46 (< .001
PI (N*s)	148 ± 42.8	130 ± 34*^†^	155 ± 41.3	137 ± 42*^§^	152 ± 40.4	142 ± 37.9	154 ± 42.5	146 ± 41	**14.53 (.002)**	66.61 (< .001)	5.69 (.128)
RMP (W/Bw)	41.8 ± 9.11	36.2 ± 5.81*	43.7 ± 7.36	38.2 ± 8.01*	43.2 ± 7.63	39.7 ± 6.26	42.7 ± 7.4	39.9 ± 7.38	**8.53 (.036)**	78,84 (< .001)	6.63 (.084)
AMP (W)	2789 ± 945	2416 ± 720*^†^	2979 ± 948	2609 ± 905*^§^	2949 ± 931	2683 ± 829	2944 ± 943	2752 ± 915*	**9.57 (.023)**	84.97 (< .001)	15.88 (.001)
MP (W)	1382 ± 450	1144 ± 339*^†^	1450 ± 469	1240 ± 403*	1499 ± 457	1346 ± 406*	1503 ± 455	1353 ± 438*	**8.32 (.040)**	91.88 (< .001)	28.05 (< .001)
RFD_Max_	6026 ± 2039^†^	5270 ± 2351*	6033 ± 2589^§^	5203 ± 2181*^§^	6521 ± 2695	5857 ± 2575*	6514 ± 2460	6000 ± 2508*	**71.3 (< .001)**	174.4 (< .001)	66.7 (< .001)

Significant values are in [bold].

*RFM* relative maximal force, *AMF* absolute maximal force, *MF* mean force, *PPI* positive push impulse, *PI* push impulse, *RMP* relative maximal power, *AMP* absolute maximal power, *MP* mean power, *RFD*_*Max*_ maximum value of rate of force development, *P inter P* value for interaction.

*P* value on the table is related to factor interaction.

*Difference between Pre and post.

^#^Difference with A1.

^†^Difference with A2.

^Ψ^Difference with G1.

^§^Difference with G2.

**Fig. 3:**
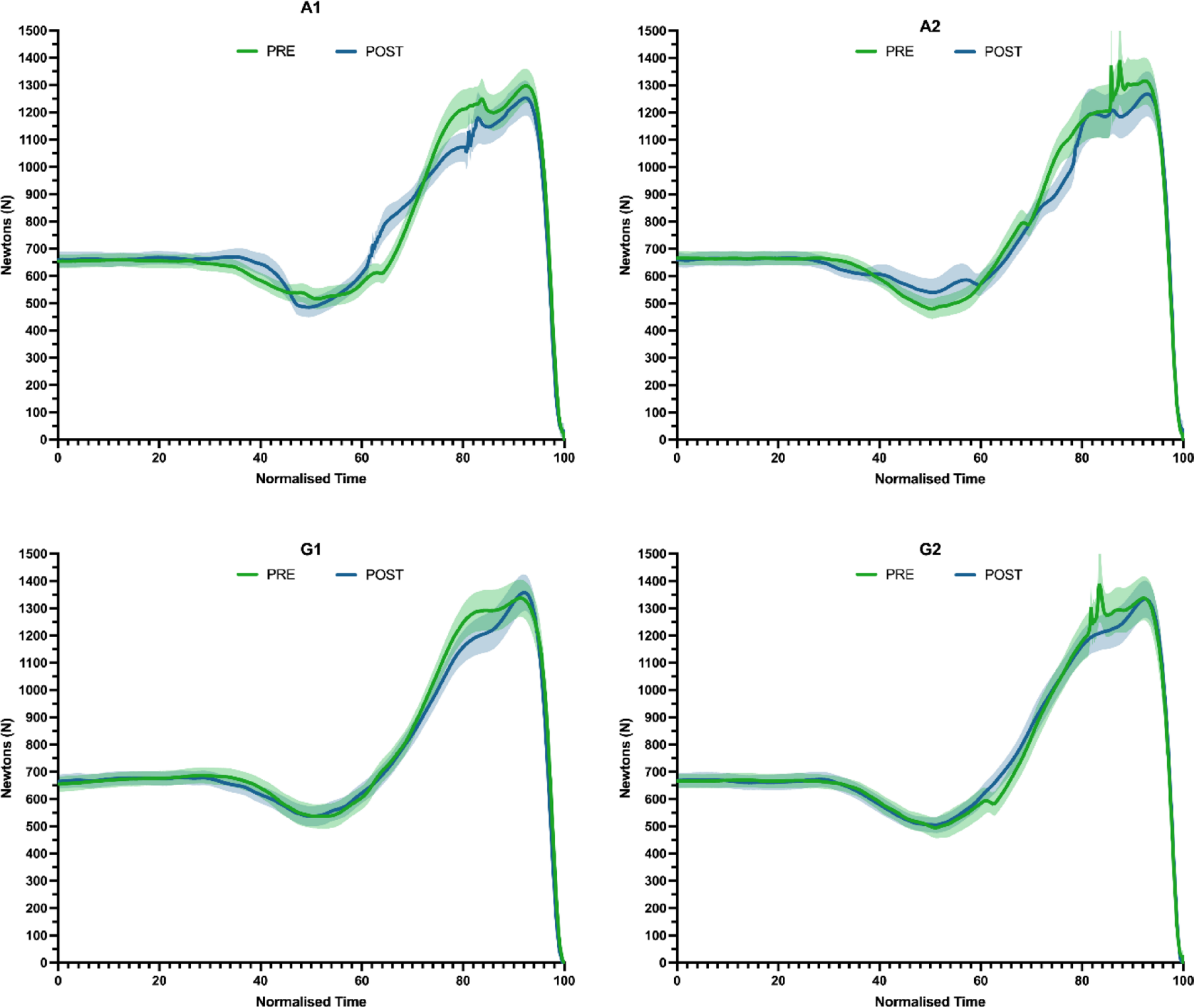
Graphical evolution of the CMJ recorded Pre- and Post-intervention, according to the groups and the series carried out. The data were standardized to facilitate analysis and graphical representation. Note: A1: Alternated 1; A2: Alternated 2; G1: Grouped 1; G2: Grouped 2.

The comparison between CMJ performances as per measurement time and group is shown in Fig. 3. Force–time curves reveal that both A1 and G1 conditions exhibit a diminished eccentric peak of force at around 80% normalized time in the post-intervention measurements. This reduction is less evident in A2 and G2 conditions. However, there are no notable differences in the maximal concentric force across the conditions. Furthermore, A2, and especially A1, exhibit a reduction in the eccentric RFD in subsequent assessments, indicating a general trend where participants require a longer duration to develop the same level of force in the eccentric phase after the intervention.

A two-way ANOVA was conducted to examine the effects of time and group on blood lactate concentration. The analysis revealed a statistically significant interaction between time and group (*F*_(6, 346)_ = 20.07; *p* < .01; *η*_*p*_^2^ = 0.258). Further examination of simple main effects indicated that both time (*F*_(2, 346)_ = 405.8; *p* < .01; *η*_*p*_^2^ = 0.701) and group (*F*_(3, 346)_ = 49.89; *p* < .01; *η*_*p*_^2^ = 0.302) independently exerted significant effects on blood lactate concentration.

**Fig. 4:**
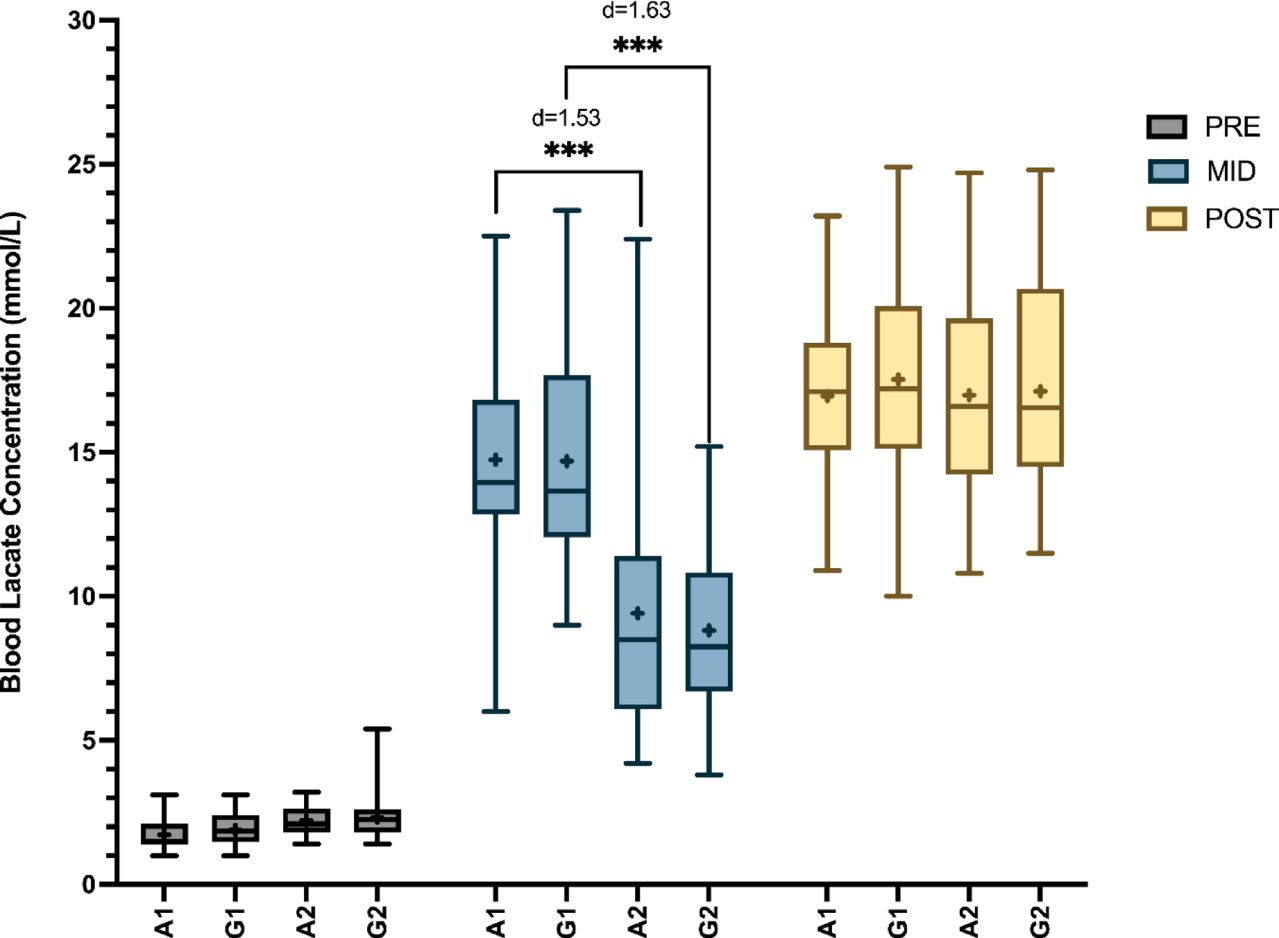
Changes in blood lactate concentration. Note: A1: Alternated 1; A2: Alternated 2; G1: Grouped 1; G2: Grouped 2; Sig Codes: ***p < .001.

In the Pre, blood lactate concentrations were low and consistent across all groups, with no significant differences observed, with all circuits showing values ~ 2 mmol/L. In the Mid measurements statistical analysis revealed significant differences practically between all groups (*p* < .001). Specifically, groups A1 and G1 (groups with the maximal number of repetitions performed during the two laps) exhibited significantly higher lactate levels compared to groups A2 (*p* < .001; *d* = 1.53; 95% CI [0.95–2.11]) and G2 (*p* < .001; *d* = 1.63; 95% CI [1.05–2.21]) respectively. Finally, Post measurements did not show significant differences between the circuit’s interventions with values ranging from ~ 17–18 mmol/L. The differences between the groups and the timing of the measurements are illustrated in Fig. 4.

**Fig. 5:**
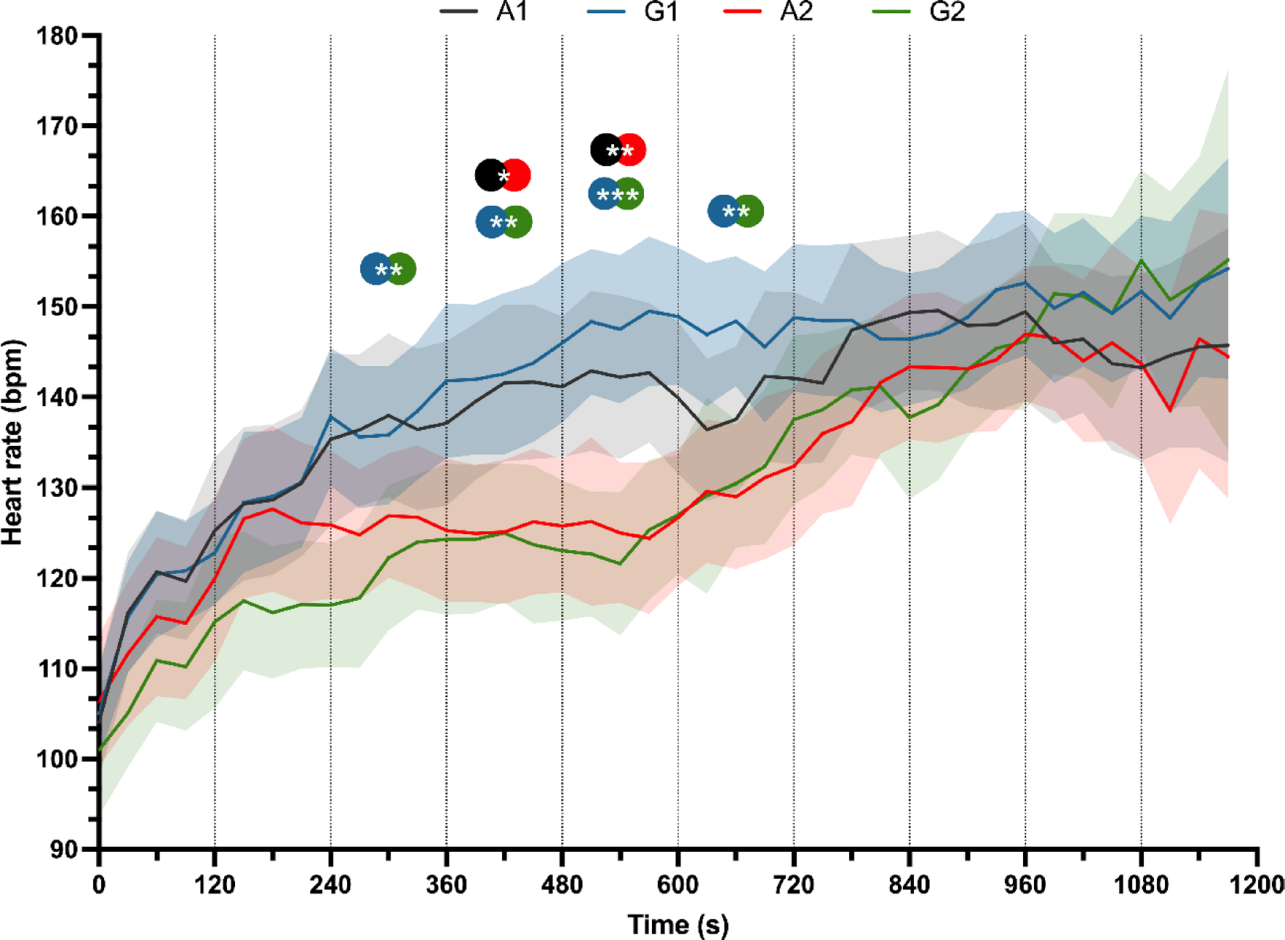
Heart Rate across the time during the exercise. Black represents group A1, Red for A2, Blue represents G1 and Green for G2 groups. Sig codes: *p < .05; **p < .01; ***p < .001.

Moreover, Fig. 5 illustrates the heart rate (HR) responses over a 20-min period across four distinct circuit training modalities. A one-way ANOVA was conducted to evaluate the effect of circuit training structure on heart rate at various time intervals. During the initial phase (0–240 s), all groups exhibited a gradual increase in HR from approximately 100 beats per minute (bpm) to 130–140 bpm, with no statistically significant differences observed between groups.

However, significant differences emerged in subsequent intervals: between 240 and 360 s, (*F*_(3, 64.4)_ = 5.396; *p* = .002; *η*_*p*_^2^ = 0.119); 360–480 s (*F*_(3, 64,3)_ = 6.190; *p* < .001; *η*_*p*_^2^ = 0.140); 480–570 s (*F*_(3, 64,3)_ = 11.008; *p* < .001; *η*_*p*_^2^ = 0.223); and 600–690 s (*F*_(3, 64,3)_ = 5.689;* p* = .002; *η*_*p*_^2^ = 0.125). Post-hoc analyses indicated that differences began to appear in the 240–360 s interval, with group G2 demonstrating significantly lower HR compared to G1 (*p* < .001; *d* = 0.93; 95% CI [0.326, 1.373]). Between 360 and 450 s, significant differences were observed between A1 and A2 (*p* = .038; *d* = 0.68; 95% CI [0.180, 1.219]), and between G1 and G2 (*p* = .005; *d* = 0.90; 95% CI [0.350, 1.345]). These differences persisted during the 480–570 s interval, with statistically significant contrasts between A1 and A2 (*p* = .008; *d* = 0.82; 95% CI [0.314, 1.360]), and between G1 and G2 (*p* < .001; *d* = 1.22; 95% CI [0.689, 1.760]). During the 600–690 s interval, significant differences remained only between G1 and G2 (*p* = .005; *d* = 0.87; 95% CI [0.352, 1.399]). No statistically significant differences were detected among groups following the 720-s mark.

## Discussion

This research aimed to analyse the physiological and mechanical responses to different structures of circuit resistance training in young adults. According to the findings of this study, the different exercise configurations (grouped and alternated) were not able to significantly modulate mechanical or physiological responses, although differences in volume led to notable changes in the results.

Specifically, the vertical jump had its PI, AMP, MP, and RDF_Max_ properties modulated, but its loss percentage of jump height was not modified by the exercise structures. However, the protocols with higher volume (A1 and G1) result in greater decreases in jump height compared to those with lower volume (A2 and G2). Thus, training volume, rather than its organisation appears to be the variable with the greatest influence on neuromuscular fatigue resulting in CMJ decreasement performance. Previous research supports that intense, high-volume training sessions can cause central and peripheral fatigue, diminishing the efficiency of the neuromuscular system in short-duration movements^24^.

Our results are in harmony with the research of Correa Neto et al.^18^, where small differences between the traditional and alternated training groups were noted for the rate of perceived exertion, and the influence of the volume of repetitions was highlighted to explain these differences. Concerning the exercise structures, the study by Pirauá et al.^25^ found no significant differences in total workload or the rate of perceived exertion, although in their research an alternating structures of exercises is permanently used, turning the order as a modification. However, some studies have shown an influence on strength or stability gains when using circuit resistance training considering the organisation of the exercises when protocols are extended by 8–10 weeks^15,26^. Nevertheless, the samples selected in that research were composed of untrained individuals, which could influence the results due to the training effect.

Additionally, the analysis of force–time curves highlights significant post-intervention modifications, particularly in the eccentric phase of force production. The reduction in eccentric peak force and RFD in conditions A1 and G1 suggests that while participants can still achieve the same absolute force levels, the efficiency of force development could be compromised, requiring a longer duration. The overall similarity in eccentric force trends suggests that the intervention uniformly impacts the eccentric phase across different circuit designs. This could affect the jump height achieved in CMJ^27^ as the absolute and relative peak of eccentric force seems to be closely related to the performance in this jump^28^. This could suggest fewer active actin-myosin cross-bridges, decreasing the potential for eccentric force production^28^. This might lead to adaptations in neuromuscular coordination or changes in muscle–tendon unit stiffness post-intervention. In contrast, no change in the maximal concentric force has been detected, suggesting that the concentric phase remains could be unaffected by the intervention, with the impact being primarily focused on the eccentric component of force production. Nevertheless, from a descriptive approach, the alternated circuits (A1 and A2) exhibit lower concentric peak forces compared to the grouped circuits (G1 and G2), indicating a potential effect in concentric force generation. This disparity suggests that the alternated circuits can produce lower effects in the decreasement of concentric strength.

Jump loss has been positively correlated with other markers such as lactate in establishing fatigue-related measurements^29^. The results showed that all groups began with comparable blood lactate concentrations, establishing a common baseline. However, there was a significant increase in blood lactate levels across all groups, suggesting a strong physiological response to the intervention. The significant differences between groups suggest varied responses, likely due to variations in the volume of the first lap. This indicates that exercise order may not significantly impact metabolic stress, as reflected by the lactate response. However, intervention volume appears to have a substantial effect on mid-session measurements. It is important to note that while all groups performed similar volumes during the second lap, participants in A2 and G2 completed only 50% of their maximal repetitions in the first lap. This difference in accumulated volume may explain the variations observed in the lactate response. Given the increase in the number of repetitions, it is understandable that such differences may arise^30^, although they could also be influenced by mechanical variables^31^. Interestingly, these differences do not persist in the post-intervention measurements. The lack of significant differences between groups at this stage might suggest that the initial varied responses converged over time.

Furthermore, heart rate has historically been used as another indicator of exercise intensity due to the relatively linear relationship between HR and VO2 or exercise intensity^32,33^. In this context, the heart rate data indicate that the intervention elicits a significant cardiovascular response, varying in intensity across groups, particularly evident in the mid-measurements that are influenced by the intervention volume. Similar to the lactate response, HR is significantly impacted only in interventions with lower volume during the first lap. However, during the second lap, where all interventions involved maximal repetitions, no differences were observed between the groups. Additionally, no effect of exercise order on HR was found across the interventions. Although there is evidence of the benefits of this type of training on cardiovascular variables, both during the intervention^9,34^ and in its subsequent effects^35^, to the best of our knowledge, this is the first study that graphs, tracks and report the evolution of heart rate throughout this type of training. This highlights the differences that could arise from training volume, which might be more complex to identify using absolute values alone. In addition, although acute cardiovascular responses were observed through heart rate monitoring during the circuit training sessions, it is important to clarify that these responses should not be interpreted as equivalent to the physiological adaptations typically associated with aerobic exercise (aerobic capacity, cardiac function or central cardiovascular adaptations). The heart rate elevations recorded in this study represent the acute response induced by the configuration and volume of the resistance circuits.

These findings provide valuable insights into the physiological demands of the intervention and underscore the importance of considering individual differences in designing and evaluating exercise programs. The importance of training volume as a key feature of training fatigue in circuits appears to have a greater impact than the way training is organised.

### Limitations and recommendations for further research

This study provides valuable insights into the acute physiological and mechanical responses to different circuit resistance training configurations, using a robust within-participants design and a combination of metabolic and neuromuscular markers. However, several limitations must be acknowledged. First, while the sample included both male and female participants, the menstrual cycle phase of the female participants was not monitored, which may have influenced certain physiological responses. Second, nutritional intake, hydration status, and supplementation were not standardized across sessions, which could have introduced variability in fatigue and lactate responses. Third, while the selected mechanical and physiological variables offer meaningful indicators of internal load, fatigue is inherently multifactorial, and other dimensions such as psychological stress, hormonal responses, or neuromuscular coordination were not assessed. Despite these limitations, the use of standardized exercise protocols, objective mechanical data, and continuous heart rate monitoring strengthens the overall internal validity of the study. Given the complexity of fatigue as a physiological and psychological phenomenon, future research should adopt a more multifaceted approach that includes additional markers such as perceived exertion, hormonal profiles, blood biomarkers, and cognitive or motor performance indicators. Moreover, nutritional status, hydration, and supplementation should be controlled or at least recorded to better isolate the effects of training variables. Importantly, future studies should consider analyzing sex-specific responses independently, as hormonal fluctuations, particularly in female athletes, may differentially influence fatigue, recovery, and metabolic stress.

## Conclusion

In conclusion, the study found that volume was the key factor influencing the differences in physiological and mechanical responses between the groups. Higher-volume circuits led to greater reductions in power-related variables and more pronounced impacts on the rate of force development. Additionally, blood lactate levels and heart rate varied significantly based on volume, highlighting the importance of carefully managing volume in circuit resistance training to achieve specific training outcomes. However, seems to be no effects between alternating and grouped exercise arrangements, indicating that the impact is due to volume rather than the exercise structures.

### Practical applications

As practical applications, movement professionals should adjust the volume of circuit training based on individual goals, using it as a key variable to target specific physiological responses, such as RFD or cardiovascular stress. When higher-volume circuits are chosen, which may lead to greater fatigue and reductions in RFD, it is necessary to integrate longer rest periods or lower-intensity sessions between high-volume training days to optimize recovery and maintain performance.

## Data Availability

The raw data supporting the conclusions of this article will be made available by the authors, without undue reservation, through contact with the correspondent author (*).
